# Differential patterns of connectivity in Western Pacific hydrothermal vent metapopulations: A comparison of biophysical and genetic models

**DOI:** 10.1111/eva.13326

**Published:** 2021-12-09

**Authors:** Corinna Breusing, Shannon B. Johnson, Satoshi Mitarai, Roxanne A. Beinart, Verena Tunnicliffe

**Affiliations:** ^1^ Graduate School of Oceanography University of Rhode Island Narragansett Rhode Island USA; ^2^ Monterey Bay Aquarium Research Institute Moss Landing California USA; ^3^ Okinawa Institute of Science and Technology Graduate University Kunigami‐gun Japan; ^4^ Department of Biology School of Earth and Ocean Sciences University of Victoria Victoria British Columbia Canada

**Keywords:** biophysical modelling, deep‐seabed mining, gene flow, hydrothermal vents, meta‐population connectivity

## Abstract

Hydrothermal ecosystems face threats from planned deep‐seabed mining activities, despite the fact that patterns of realized connectivity among vent‐associated populations and communities are still poorly understood. Since populations of vent endemic species depend on larval dispersal to maintain connectivity and resilience to habitat changes, effective conservation strategies for hydrothermal ecosystems should include assessments of metapopulation dynamics. In this study, we combined population genetic methods with biophysical models to assess strength and direction of gene flow within four species of the genus *Alviniconcha* (*A*. *boucheti*, *A*. *kojimai*, *A*. *strummeri* and *A*. *hessleri*) that are ecologically dominant taxa at Western Pacific hydrothermal vents. In contrast to predictions from dispersal models, among‐basin migration in *A*. *boucheti* occurred predominantly in an eastward direction, while populations within the North Fiji Basin were clearly structured despite the absence of oceanographic barriers. Dispersal models and genetic data were largely in agreement for the other *Alviniconcha* species, suggesting limited between‐basin migration for *A*. *kojimai*, lack of genetic structure in *A*. *strummeri* within the Lau Basin and restricted gene flow between northern and southern *A*. *hessleri* populations in the Mariana back‐arc as a result of oceanic current conditions. Our findings show that gene flow patterns in ecologically similar congeneric species can be remarkably different and surprisingly limited depending on environmental and evolutionary contexts. These results are relevant to regional conservation planning and to considerations of similar integrated analyses for any vent metapopulations under threat from seabed mining.

## 1 INTRODUCTION

Connectivity, the exchange of individuals among spatially separated populations is a critical concept in marine conservation biology because of its role in maintaining genetic diversity and in enhancing the resilience of metapopulations to environmental disturbances (Botsford et al., 2001; Cowen et al., 2007; Cowen & Sponaugle, 2009). Effective connectivity is achieved when individuals disperse from one population to another, then successfully recruit and reproduce at the recipient site. In the benthic marine environment, dispersal typically occurs by larval stages that must either feed on plankton or obtain nutrition from internal energy reserves to complete metamorphosis. Planktotrophy is often associated with longer pelagic larval durations (PLD) and increased dispersal potential, while lecithotrophy is usually correlated with shorter PLDs and smaller dispersal distances (Levin, 2006). However, this relationship is not universal, and factors such as larval supply do not always predict recruitment (Pineda et al., 2010). For example, dispersal‐unrelated ecological factors, such as differential habitat selection, environmental cues and interspecific competition, can affect recruitment success of settling larvae, thereby disconnecting dispersal from connectivity (Jenkins et al., 2009). Due to the difficulty of monitoring dispersal and recruitment events at sea, a variety of indirect methods can inform connectivity between populations, including biophysical models, geochemical tracers and genetic markers (Cowen & Sponaugle, 2009). While biophysical models are often useful to assess dispersal distances and patterns, genetic markers provide estimates of recruitment success and, thus, realized gene flow within a metapopulation. With respect to marine ecosystem management, this information can be integrated to identify crucial source populations for conservation and to define the sizes and spacing of marine protected areas (Boschen et al., 2016).

Human activities impact marine ecosystems in a variety of ways, including pollution, habitat destruction, overexploitation and climate change (Halpern et al., 2008, 2015; Hoegh‐Guldberg & Bruno, 2010; Levin et al., 2020). In the deep sea, proposed mining of polymetallic seafloor massive sulphide (SMS) deposits is currently the most acute threat to hydrothermal vent communities worldwide (Blasiak et al., 2020; Miller et al., 2018; Van Dover et al., 2018). While the full impact of mining is unknown, the main anticipated risks are changes in seafloor and water properties, adulteration of surrounding ecosystems through sediment plumes and irreversible habitat and biodiversity loss (Drazen et al., 2020; Levin et al., 2020; Miller et al., 2018; Niner et al., 2018). Back‐arc basins, which are seafloor spreading centres that form behind volcanic arcs at subduction zones, often contain SMS deposits with particularly high metal grades (Petersen et al., 2016), and exploration tenement blocks for these deposits were issued by several island states. However, a 10‐year moratorium on mining proposed for Southwest Pacific countries was enacted by a few states until a better understanding emerges of environmental and societal impacts (Kakee, 2020). Such a moratorium, more broadly applied, could provide important opportunities to improve knowledge on hydrothermal vent communities, including connectivity patterns among Western Pacific vent populations, to help inform management plans for conservation.

Our current understanding of dispersal and connectivity in the West Pacific is based on a growing number of population genetic and biophysical modelling studies (Mitarai et al., 2016; Plouviez et al., 2019; Thaler et al., 2011, 2014; Yearsley & Sigwart, 2011). Using estimated PLDs in the water column and oceanographic current information collected with autonomous profiling floats, Mitarai et al. (2016) developed a dispersal model for depths between 100 and 1500 m among West Pacific hydrothermal vent locations. Results from these modelling analyses indicate that ocean circulation patterns should allow for widespread bidirectional dispersal within basins, but largely restrict basin‐to‐basin transport. While the southern Mariana region appeared to be virtually isolated, modelled dispersal among the Lau, North Fiji and Manus basins occurred exclusively in a westward direction via intermediate stepping stone sites. Limited bidirectional connections were only observed between the North Fiji Basin and New Hebrides Volcanic Arc (Vanuatu).

Several population genetic studies have supported the predictions from Mitarai et al.'s (2016) model, implying significant population genetic subdivision and limited migration among disparate back‐arc basins for many vent‐associated species (Breusing, Johnson, et al., 2020; Kojima et al., 2000; Kyuno et al., 2009; Thaler et al., 2011, 2014). By contrast, some molecular studies provide evidence for eastward‐directed gene flow between Southwest Pacific back‐arc basins (Plouviez et al., 2019; Thaler et al., 2011) and restricted gene flow within basins (Breusing, Johnson, et al., 2020; Thaler et al., 2014), suggesting that complementary genetic analyses are needed to capture the full range of larval dispersal scenarios and realized connectivity between vent populations in this region.

As a key foundation taxon mostly associated with active sulphide chimneys, deep‐sea snails of the genus *Alviniconcha* (Gastropoda; Abyssochrysoidea) form important biomass and community relationships at West Pacific vents (Desbruyères et al., 1994; Sen et al., 2013). Similar to most other dominant vent‐dwelling invertebrate species, *Alviniconcha* snails establish endosymbioses with chemosynthetic, environmentally acquired bacteria that derive energy from the oxidation of reduced sulphur compounds or hydrogen to produce nutrition for their hosts (Beinart et al., 2012, 2015; Breusing, Mitchell, et al., 2020; Henry et al., 2008; Suzuki et al., 2005, 2006; Urakawa et al., 2005). The genus has diversified into six known extant lineages that are subdivided across the Indo‐Pacific Ocean (Breusing, Johnson, et al., 2020; Johnson et al., 2015). In the Western Pacific, four species of *Alviniconcha* are currently recognized to inhabit deep vent sites (>1000 m) at back‐arc spreading centres and volcanic arcs (Breusing, Johnson, et al., 2020; Johnson et al., 2015). *Alviniconcha kojimai* is known from the Manus, Lau and North Fiji basins; *A*. *boucheti* is co‐distributed with *A*. *kojimai* but also occurs at volcanoes of the Vanuatu island arc, the Tonga volcanic arc and American Samoa, spanning a range of approximately 4000 km. *Alviniconcha strummeri* occurs predominantly in the Lau Basin, but has recently been collected from the North Fiji Basin and Futuna (D. Jollivet, personal communication). Lastly, in the Northwest Pacific, *A*. *hessleri* inhabits vents along 600 km of the Mariana back‐arc spreading centre. The larval biology of all *Alviniconcha* species is largely unknown, although a planktotrophic development has been suggested based on examinations of protoconch characteristics (Warén & Bouchet, 1993). Given that larval stages of one *Alviniconcha* species have been detected in the upper mesopelagic (between 100 and 200 m) (Sommer et al., 2017), it is likely that larvae can vertically migrate, thereby increasing potential for long‐distance dispersal. Due to the ecological significance and widespread distribution of this genus, knowledge of connectivity patterns between *Alviniconcha* populations should help to predict effects of anthropogenic disturbances, especially in the context of source and sink locations (Boschen et al., 2016). Such insights are also urgently needed as all *Alviniconcha* species, like some other vent endemic taxa, have been classified as endangered or vulnerable by the IUCN (https://www.iucnredlist.org/).

We used population genetic analyses and isolation with migration models to determine patterns of intraspecific gene flow within and among back‐arc basins in the western Pacific species *A*. *boucheti*, *A*. *kojimai*, *A*. *strummeri* and *A*. *hessleri*. We then compared strengths and directions of gene flow with dispersal probabilities from biophysical modelling to test whether these models are good proxies for connectivity in this important genus.

## 2 MATERIALS AND METHODS

### 2.1 Genetic analyses

Previously published DNA sequences from Johnson et al. (2015) and Breusing, Johnson, et al. (2020) for fragments of one mitochondrial gene (cytochrome‐*c*‐oxidase subunit I, mt*COI*) and three nuclear genes (elongation factor 1 alpha, *EF1a*; ATP synthetase subunit alpha, *ATPSa*; ATP synthetase subunit beta, *ATPSb*) were used to investigate gene flow among populations of *A*. *boucheti*, *A*. *kojimai*, *A*. *strummeri* and *A*. *hessleri* (Table 1). Genetic differentiation between populations was assessed by calculating pairwise *F*_ST_ values with the diversity package in R (Keenan et al., 2013; R Core Team, 2018) based on the method by Weir and Cockerham (1984). Variance partitioning between hierarchical levels of genetic subdivision was inferred through Analysis of Molecular Variance with poppr (Kamvar et al., 2014). Diversity statistics for each gene and species, including nucleotide diversity (*π*), haplotype diversity (*H*) and number of segregating sites (*S*), were computed with the R packages ape (Paradis & Schliep, 2019) and pegas (Paradis, 2010). To determine departures from selective neutrality of each genetic marker within species, we performed the DHEW compound test (Zeng et al., 2007; https://github.com/drkaizeng/publications‐and‐software) and the multilocus HKA test (Wright & Charlesworth, 2004; https://github.com/rossibarra/MLHKA). Significance of the DHEW test was assessed through 50,000 coalescent simulations at a *p* value of 0.05. The MLHKA test was run with 500,000 MCMC iterations and repeated three times with different random number seeds. A random sequence of *A*. *boucheti* for each gene was used as outgroup for analyses in *A*. *kojimai* and *A*. *strummeri*, while *A*. *kojimai* and *A*. *strummeri* were used as outgroups for analyses in *A*. *boucheti* and *A*. *hessleri* respectively. Significance was assessed through log likelihood ratio tests at a *p* value threshold of 0.05. Both the DHEW and MLHKA tests are relatively robust to demographic history and should thus be more powerful in detecting signatures of selection than traditional methods (Wright & Charlesworth, 2004; Zeng et al., 2007). We considered the consensus of both methods as evidence for positive selection.

**TABLE 1:** Sampling information for *Alviniconcha* species investigated in this study

Dive #	Locality	Abbr.	Latitude	Longitude	Depth (m)	Year	*A. boucheti*	*A. kojimai*	*A. strummeri*	*A. hessleri*
Lau Basin							40/23/19/34	58/21/33/51	56/34/46/50	
J2‐140/141	Kilo Moana	KM	20° 03.222' S	176° 08.009' W	2620	2005	29/14/19/27	–	–	–
J2‐142	Tow Cam	TC	20° 19.076' S	176° 08.258' W	2714	2005	–	13/5/12/13	–	–
R‐1933			20° 19.000' S	176° 08.204' W	2705	2016				
R‐1932/1935	Tahi Moana	THM	20° 40.409' S	176° 10.848' W	2273–2280	2016	–	4/0/1/3	22/12/19/23	–
R‐1922/1931	ABE	ABE	20° 45.700' S	176° 11.500' W	2130–2155	2016	11/9/0/7	3/0/0/1	–	–
J2‐143/144	Tu'i Malila	TM	21° 59.431' S	176° 34.146' W	1845	2005	–	38/16/20/34	34/22/27/27	–
R‐1924–1930			21° 59.347' S	176° 34.092' W	1883–1889	2016				
Tonga Volcanic Arc							19/16/3/9			
R‐1918/1919	Niua South	NS	15° 09.885' S	173° 34.468' W	1156–1164	2016	19/16/3/9	–	–	–
North Fiji Basin							47/25/26/39	9/2/2/3		
J2‐149/150	White Lady	WL	16° 59.398' S	173° 54.953' E	1970	2005	17/5/12/16	1/0/1/1	–	–
J2‐151/152	Mussel Hill	MH	16° 59.410' S	173° 54.970' E	1973	2005	15/6/8/9	4/0/0/0	–	–
J2‐153	White Rhino	WR	16° 59.446' S	173° 54.862' E	1978	2005	15/14/6/14	4/2/1/2	–	–
Vanuatu (New Hebrides Arc)							6/5/5/3			
KI‐27/60	Nifonea	NF	18° 08.000' S	169° 31.000' E	1900	2013	6/5/5/3	–	–	–
Manus Basin							84/49/52/52	53/23/37/38		
ST‐28/30	Solwara 8‐2	SW8	3° 43.824' S	151° 40.458' E	1710	2008	18/14/13/14	1/0/1/1	–	–
ST‐9/11/17	Solwara 1	SW1	3° 47.436' S	152° 05.472' E	1480–1530	2008	25/16/19/17	52/23/36/37	–	–
			3° 47.370' S	152° 05.778' E						
			3° 47.370' S	152° 05.616' E						
ST‐38/40	South Su	SS	3° 48.564' S	152° 06.144' E	1300–1350	2008	41/19/20/21	–	–	–
			3° 48.492' S	152° 06.186' E						
Mariana Back‐Arc										91/50/60/59
J2‐42	Snail Site	SN	12° 57.250' N	143° 37.200' E	2863	2003	–	–	–	23/24/19/27
J2‐185/S‐185	Forecast	FC	13° 23.680' N	143° 55.207' E	1447–1475	2006	–	–	–	10/11/14/15
Su‐47	Perseverance	PS	15° 28.810' N	144° 30.462' E	3909	2016	–	–	–	6/0/2/3
Su‐41/44	Hafa Adai	HA	16° 57.669' N	144° 52.017' E	3274–3278	2016	–	–	–	18/5/7/3
Su‐40	Burke	BU	18° 10.954' N	144° 43.193' E	3631	2016	–	–	–	4/0/2/0
Su‐39	Alice Springs	AS	18° 12.619' N	144° 42.438' E	3611	2016	–	–	–	23/9/13/8
S‐140/141			18° 12.800' N	144° 42.400' E	3589	1992				
Su‐37	Illium	IL	18° 12.815' N	144° 42.450' E	3582	2016	–	–	–	7/1/3/5

Note

Numbers in species columns indicate samples sequenced for mt*COI*, *ATPSa*, *ATPSb* and *EF1a* respectively (Total sequences: *A*. *boucheti* = 196/118/105/137; *A*. *kojimai* = 120/46/72/92; *A*. *strummeri* = 56/34/46/50; *A*. *hessleri* = 91/50/60/59). Submersibles: J2 = *Jason II*, S = *Shinkai 6500*, Su = *SuBastian*, R = *Ropos*, ST = ST212 and KI = *Kiel 6000*.

To assess patterns of contemporary and historical migration, we used Bayesian inference implemented in Ima3 v.1.11 (Hey et al., 2018). Intraspecific gene flow analyses were performed among basins for *A*. *boucheti* and *A*. *kojimai* and within basins for *A*. *strummeri* and *A*. *hessleri*. For *A*. *boucheti*, we particularly estimated migration into and out of Niua South (Tonga volcanic arc), as biophysical models predict that this locality acts as an important stepping stone and source site for dispersal (Mitarai et al., 2016). To ensure convergence of parameters, we pooled populations by basin for *A*. *boucheti* and *A*. *kojimai* and grouped populations into three latitudinal clusters for *A*. *hessleri* (South = Forecast, Snail; Mid‐latitude = Hafa Adai, Perseverance; North = Alice Springs, Illium, Burke). Gene regions that showed evidence for intralocus recombination based on the four‐gamete test (Woerner et al., 2007) were excluded from analysis. Runs included 8–25 MCMC chains (–hn) with geometric heating (–ha: 0.99; –hb: 0.5), migration prior values (–*m*) of 10–100, a maximum population size (–*q*) of 100–250 and a maximum time of population splitting (–*t*) of 5–50. All analyses were run for at least 10^7^ generations after a burn‐in of 10^4^ generations. All loci were used for analyses, although neutrality tests indicated signatures of selection in some of the markers (see Section 3). Strasburg and Rieseberg (2010) showed that parameter estimates are relatively robust to small and moderate violations of the IM model, as is often encountered in natural populations. We are therefore confident that the inclusion of non‐neutral loci has little effect on the overall results.

### 2.2 Biophysical modelling

Particle tracking analyses generally followed Mitarai et al. (2016) based on three‐dimensional velocities simulated with the most recent version of the Regional Ocean Modeling System for the Western Pacific at a 1–5 km mesh resolution (Shchepetkin, 2015; Shchepetkin & McWilliams, 2005). Mitarai et al.'s model represents empirical data from profiling floats in back‐arc systems relatively well; it is also applicable across whole ocean basins and has sufficient temporal and spatial resolution to realistically predict dispersal scenarios. We therefore chose this modelling approach for our analyses. Probabilities for dispersal between West Pacific back‐arc basins were obtained from Mitarai et al. (2016) for dispersal cases at 500 and 1000 m depth, which are representative of the main dispersal patterns. Additional dispersal simulations were performed for the Lau Basin and Mariana back‐arc using all vent sites registered in the InterRidge v3.4 database. As in Mitarai et al. (2016), dispersal probabilities were estimated by integrating ~1 million Lagrangian, passively drifting particles that were released from each of the vent sites and tracked by accounting for temperature dependence of larval development after the unified model by O'Connor et al. (2007). This model is consistent with larval development time in hydrothermal vent barnacles (Watanabe et al., 2004) and is likely applicable to other deep‐sea vent species. Dispersal patterns at constant depths of 0, 100, 500, 1000, 1500, 2000, 2500 and 3000 m were evaluated based on the known depth distributions of *Alviniconcha* and detection of *Alviniconcha* larvae in the upper water column (Sommer et al., 2017), thereby considering potential for vertical migration. Simulated mean PLDs ranged from 18.93 ± 0.78 days (0 m) to 282.68 ± 18.86 days (3000 m). To mimic migrations over multiple generations, joint transition probabilities from source to destination sites were computed considering not only direct but also stepping stone transport, until the joint probabilities converged. All vent sites registered in the InterRidge database were included as stepping stone sites, if they were deeper than the modelled dispersal depth based on the assumption that larvae move to constant dispersal depths and detect chemical or heat anomaly cues from rising hydrothermal plumes as signal for settlement (Adams et al., 2012).

## 3 RESULTS

### 3.1 Population genetic differentiation

Populations within each species showed no significant genetic differentiation based on mitochondrial *COI*, but exhibited varying degrees of subdivision based on nuclear markers (Tables 2 and 3). *Alviniconcha boucheti* and *A*. *kojimai* were the only species that were sampled across different back‐arc basins in the Southwest Pacific.

**TABLE 2:** Genetic differentiation between *Alviniconcha boucheti*, *A*. *kojimai* and *A*. *strummeri* populations based on nuclear (upper diagonal) and mitochondrial (lower diagonal) markers

*A. boucheti*		Tonga	Lau		North Fiji			Vanuatu	Manus		
		NS	KM	ABE	WL	MH	WR	NF	SW8	SW1	SS
Tonga	NS	*	**0.1867**	*−0.0233*	**0.2893**	**0.3147**	**0.2448**	0.1039	**0.1490**	0.0806	**0.0838**
Lau	KM	−0.0289	*	*0.3497*	0.0259	0.0586	**0.3073**	**0.2033**	**0.3084**	**0.2448**	**0.2534**
	ABE	−0.0370	−0.0808	*	*0.5109*	*0.5655*	*0.4262*	*0.1257*	*0.2436*	*0.1086*	*0.1291*
North Fiji	WL	0.0457	0.0148	−0.0215	*	0.0291	**0.3897**	**0.3575**	**0.4203**	**0.3380**	**0.3509**
	MH	0.0511	0.0307	−0.0183	0.0374	*	**0.4144**	**0.3958**	**0.4613**	**0.3765**	**0.3831**
	WR	0.0601	0.0280	−0.0147	0.0290	−0.0067	*	**0.3178**	**0.4058**	**0.3306**	**0.3391**
Vanuatu	NF	−0.0384	−0.0456	−0.0905	−0.0156	0.0462	0.0714	*	−0.0054	−0.0608	−0.0594
Manus	SW8	−0.0003	−0.0053	−0.0543	0.0214	0.0283	0.0602	−0.0428	*	0.0169	0.0014
	SW1	0.0104	0.0002	−0.0450	0.0089	−0.0230	−0.0050	0.0018	−0.0063	*	0.0007
	SS	0.0082	−0.0026	−0.0644	−0.0089	0.0354	0.0376	−0.0305	−0.0129	−0.0065	*

*A. kojimai*		Lau		North Fiji	Manus	*A. strummeri*		Lau	
		TC/THM	TM	WL/WR	SW1			THM	TM
Lau	TC/THM	*	0.0703	0.1197	**0.1095**	Lau	THM	*	*0.0137*
	TM	0.0256	*	0.1154	**0.0342**		TM	−0.0103	*
North Fiji	WL/WR	0.0291	0.0441	*	0.1590				
Manus	SW1	0.0130	0.0161	0.0953	*				

Note

Significant *F*_ST_ values where bootstrapped confidence intervals did not overlap with 0 are indicated in bold. Values are italicized if confidence intervals could not be calculated.

**TABLE 3:** Genetic differentiation between *Alviniconcha hessleri* populations based on nuclear (upper diagonal) and mitochondrial (lower diagonal) markers

*A. hessleri*		North Mariana			Central Mariana		South Mariana	
		IL	AS	BU	HA	PS	FC	SN
North Mariana	IL	*	*−0.0133*	*−0.1120*	*0.0645*	*0.0192*	*0.1346*	*0.2211*
	AS	0.0908	*	*−0.0025*	0.0448	*0.0711*	**0.1754**	**0.2455**
	BU	0.0868	−0.0566	*	*0.0235*	*0.0000*	*−0.0372*	*−0.0705*
Central Mariana	HA	−0.0057	0.0177	−0.0353	*	*−0.0968*	0.0350	0.0760
	PS	0.1632	−0.0412	−0.0650	0.0360	*	*−0.0498*	*0.0074*
South Mariana	FC	0.0016	0.0073	−0.0543	−0.0312	0.0123	*	0.0987
	SN	0.0133	0.0568	−0.0406	−0.0150	0.0223	−0.0323	*

Note

Significant *F*_ST_ values where bootstrapped confidence intervals did not overlap with 0 are indicated in bold. Values are italicized if confidence intervals could not be calculated.

In *A*. *boucheti*, the Vanuatu and Manus Basin samples were significantly divergent from samples of the North Fiji Basin (*F*_ST_: 0.3178–0.4613) and the Lau Basin (*F*_ST_: 0.2033–0.3084; exception: ABE) (Table 2). Significant genetic differentiation was also detected between the Manus Basin sites (Solwara‐8 and South Su) and Niua South (*F*_ST_: 0.0838–0.1490). Among the Lau–North Fiji Basin comparisons, the only significant difference occurred between Kilo Moana and White Rhino (*F*_ST_: 0.3073). Both North Fiji Basin and Kilo Moana samples were also significantly differentiated from Niua South (*F*_ST_: 0.1867–0.3147). Within basins, no significant differentiation was observed among Manus or Lau Basin samples. By contrast, samples within the North Fiji Basin were significantly different from each other: both White Lady and Mussel Hill were strongly divergent from White Rhino despite the close proximity of these localities (*F*_ST_: 0.3897–0.4144).

In *A*. *kojimai*, significant differences were evident between the Manus and Lau Basin samples (*F*_ST_: 0.0342–0.1095), but no other significant pairwise comparisons between or within basins were observed (Table 2).

*Alviniconcha strummeri* and *A*. *hessleri* were sampled from the Lau and Mariana back‐arc spreading centres respectively. In *A*. *strummeri*, no significant differences between sampled populations were observed (Table 2). Similarly, in *A*. *hessleri*, sampled populations were undifferentiated from each other with two exceptions: the Forecast and Snail sites in the southern Mariana Basin were significantly different from the Alice Springs site in the northern Mariana Basin (*F*_ST_: 0.1754–0.2455; Table 3).

Regardless of species, most of the mitochondrial diversity was explained by variation within individuals (97.75%–100%), while less variation occurred between individuals within populations (0%–2.98%) or among broader geographic regions (0%–0.76%) (Table S1). Similarly, nuclear variation was mostly present within (24.21%–51.75%) or among individuals (40.96%–72.23%), with lower variation residing between populations (3.56%–22.50%) and regions (0%–12.95%) (Table S1).

### 3.2 Genetic diversity and neutrality analyses

Nucleotide diversity within all species was generally lower for the mitochondrial *COI* gene than for the nuclear genes (exceptions: *EF1a* in *A*. *kojimai*; *ATPSa* in *A. hessleri*), while various patterns were observed for haplotype diversity (Table S2). *H* values were consistently lower for *EF1a* compared to mitochondrial *COI*, whereas values for *ATPSa* tended to be higher in all species except for *A*. *hessleri*. *ATPSb* showed a higher haplotype diversity than mitochondrial *COI* in *A*. *boucheti* and *A*. *hessleri* but a lower diversity in *A*. *kojimai* and *A*. *strummeri*.

DHEW compound tests suggested positive selection on the mitochondrial *COI* and nuclear *ATPSb* genes in all species, while the other loci showed no significant deviation from neutrality with three exceptions (*EF1a*: *A*. *kojimai*; *ATPSa*: *A*. *kojimai*, *A*. *strummeri*; Table S2). MLHKA tests generally confirmed the results from the DHEW tests, implying selection on *COI* and *ATPSb* in *A*. *boucheti*, *A*. *strummeri* and, partly, *A*. *hessleri* (Table S3). Selection parameters for the *COI* gene were consistently smaller than 0, suggesting a reduction in polymorphism indicative of a selective sweep or purifying selection. By contrast, selection parameters for *ATPSb* were larger than 0, implying an excess of polymorphism due to balancing selection. Given that we analysed an intron of the *ATPSb* gene, it is likely that these findings represent a hitchhiking effect and no direct selection on the intron. We did not observe patterns of selection in any gene for *A. kojimai*. *EF1a* and *ATPSa* showed no deviation from neutrality in any species (exception: *ATPSa* in *A. hessleri*).

### 3.3 Isolation with migration

**FIGURE 1:**
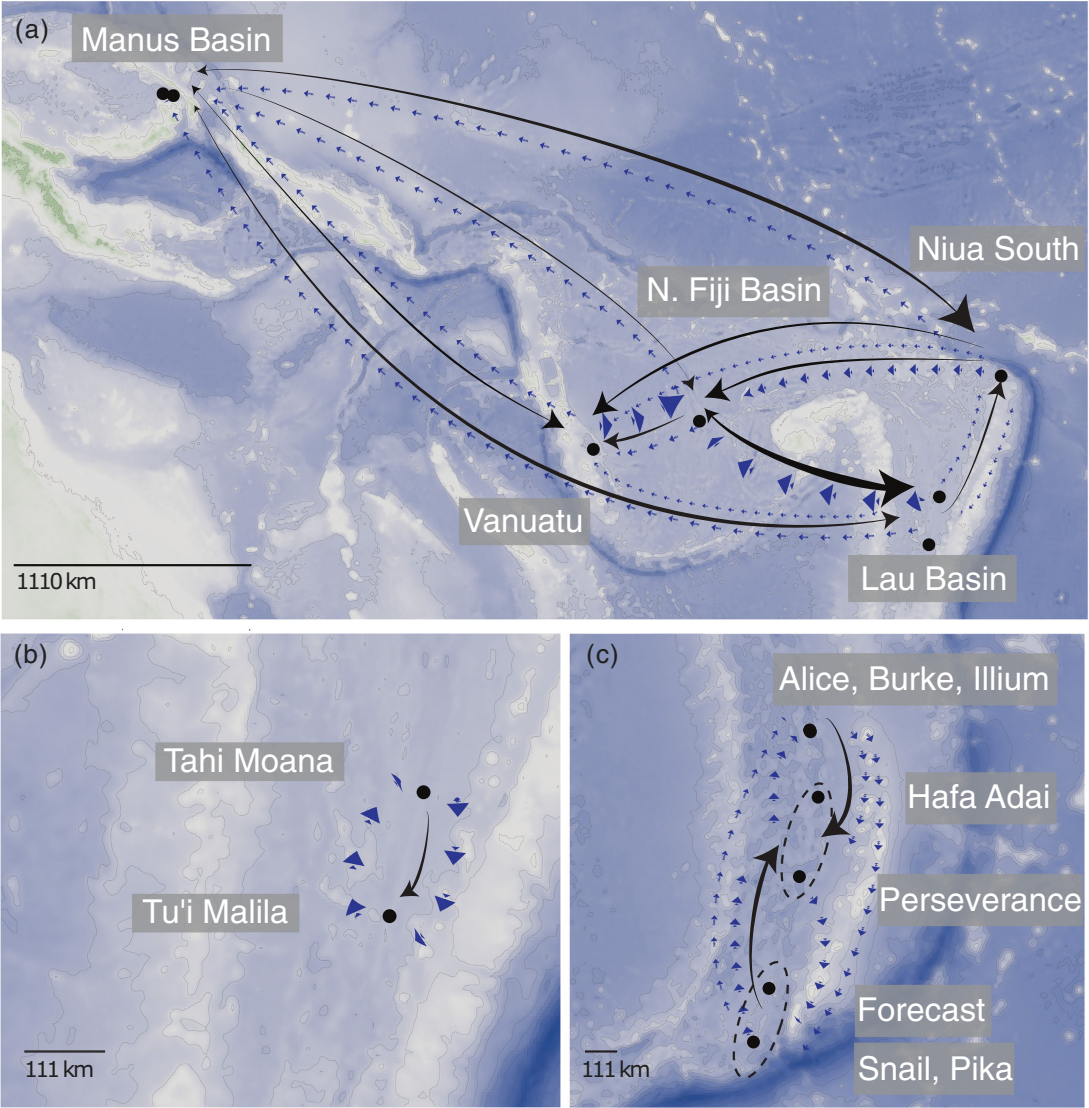
Overview of dominant migration patterns observed from genetic (black arrows) data compared to those predicted from dispersal modelling (blue arrows). (a) Migration between basins for *Alviniconcha boucheti*, (b) migration within the Lau Basin for *Alviniconcha strummeri* and (c) migration within the Mariana Back‐Arc basin for *Alviniconcha hessleri*. *Alviniconcha kojimai* did not exhibit significant gene flow between populations. Arrowhead size reflects the relative strength of dispersal potential and gene flow

Gene flow patterns were notably distinct among sympatric species in the Southwest Pacific. The predominant gene flow for *A*. *boucheti* was eastward from the Manus Basin into Niua South, Vanuatu and the Lau and North Fiji basins (Figure 1a; Table S4). Lower levels of westward migration into the Manus Basin were detected from all populations except the North Fiji Basin. Significant eastward migration was also detected from Vanuatu and the North Fiji Basin into the Lau Basin. Westward migration into the North Fiji Basin occurred from the Lau Basin and Niua South and into Vanuatu from Niua South and the North Fiji Basin. The Lau Basin populations showed significant immigration into Niua South, but we did not observe gene flow in the opposite direction. *Alviniconcha kojimai* populations appeared to be relatively isolated among basins, while *A*. *strummeri* showed only southward migration within the Lau Basin (Table S4; Figure 1b).

In the Northwest Pacific species *A. hessleri*, we found significant migration into the mid‐latitude populations Hafa Adai and Perseverance from both the northern and southern populations, but not in any other direction (Figure 1c). Ima3 allows for the inference of unsampled “ghost” populations to account for sampling gaps in the data set. We detected significant gene flow from such “ghost” populations into sampled populations in all species except for *A*. *strummeri* (Table S4).

### 3.4 Dispersal patterns based on biophysical modelling

Larval dispersal simulations implied a predominantly westward‐directed transport among Southwest Pacific basins for both analysed depths (Figure 1, Figure S1), in contrast to estimations based on genetic data. At 1000 m, the only bidirectional transport among basins was observed between Vanuatu and the North Fiji Basin (Figure S1), while additional eastward‐directed dispersal occurred at 500 m depth from Vanuatu and the North Fiji Basin to the Lau Basin and Niua South (Figure S1). In all simulations, eastward‐directed dispersal remained at a very low probability. Similarly, the probability of dispersal from any location westward to Manus Basin was <0.001 over 100 generations.

**FIGURE 2:**
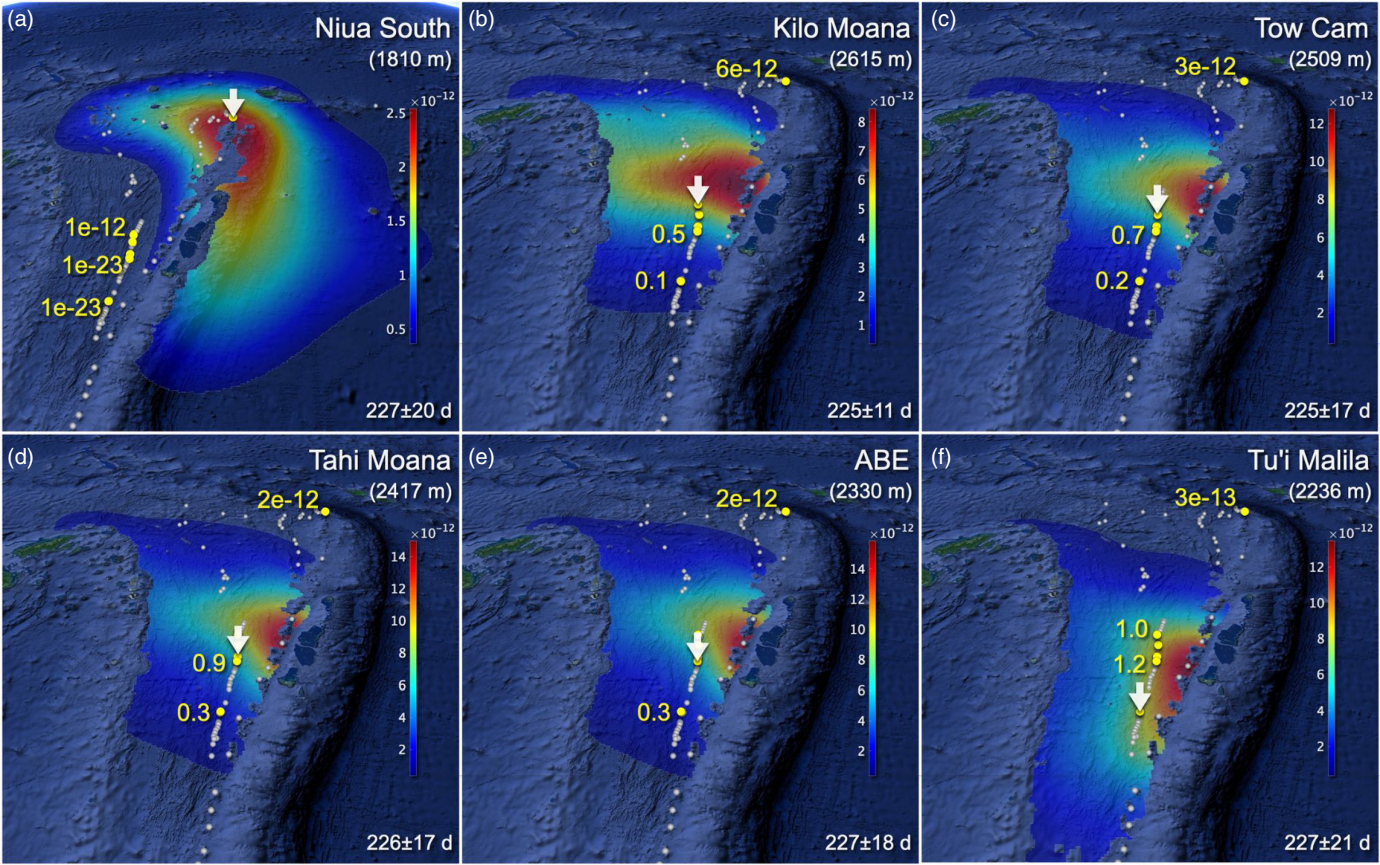
Modelled migration patterns between *Alviniconcha* populations in the Lau Basin. Colour contours indicate probability densities of water parcel displacement per unit area (square kilometres) from (a) Niua South, (b) Kilo Moana, (c) Tow Cam, (d) Tahi Moana, (e) ABE and (f) Tu'i Malila (indicated with white arrows) at a dispersal depth of 1500 m. White dots indicate all confirmed active vent sites registered in the InterRidge database version 3.4. Yellow dots show sampling sites of this study. Drift time was determined independently for each water parcel based on water temperature records and estimated metabolic rates (indicated at the bottom right corner of each panel, in days). Potential connections between the sampling sites were assessed by computing the joint transition probabilities from a source site (white arrows) to a destination site (yellow dots) via all possible paths using all active vent fields as a stepping stone (up to 100 steps). Yellow numbers indicate joint transition probabilities to destinations normalized by values to sources. Map data: Google, SIO, NOAA, U.S. Navy, NGA, GEBCO, LDEO‐Columbia, NSF

Modelled dispersal patterns within basins largely agreed with predictions from genetic migration analyses. For the Lau Basin, predicted dispersal patterns from vent locations along the Eastern Lau Spreading Center (ELSC) were very similar to each other, but contrasted to those from Niua South (Figure 2). Particles released from the ELSC sites (Kilo Moana, Tow Cam, Tahi Moana, ABE, Tu'i Malila) were mostly contained within the basin, while some exited through the northwest opening (North Fiji Passage), which qualitatively agrees with findings of the Lau Basin Float Experiment (Speer & Thurnherr, 2012). Particles from the ELSC sites also tended to move equatorward along the Tonga Ridge, as described by Simons et al. (2021). Particles from Niua South, by contrast, showed distinctive directional dispersal patterns resulting from the deep South Equatorial Current system that flows westward along the northern end of the Lau Basin and southward along the Tonga Trench. A high proportion of particles from Niua South was transported out of the basin. None of the particles from Niua South reached the ELSC sampling sites, and vice versa, for advection times of ~220 days. These sites could still be connected indirectly via stepping stone dispersal (Figure 2), although potential connections between the ELSC sites and Niua South are expected to be orders of magnitude smaller than connections among the ELSC sites. In many cases, probabilities for dispersal between the ELSC sites increased with depth, but none of the modelled dispersal depths suggested frequent connections between the ELSC sites and Niua South (Table S5).

**FIGURE 3:**
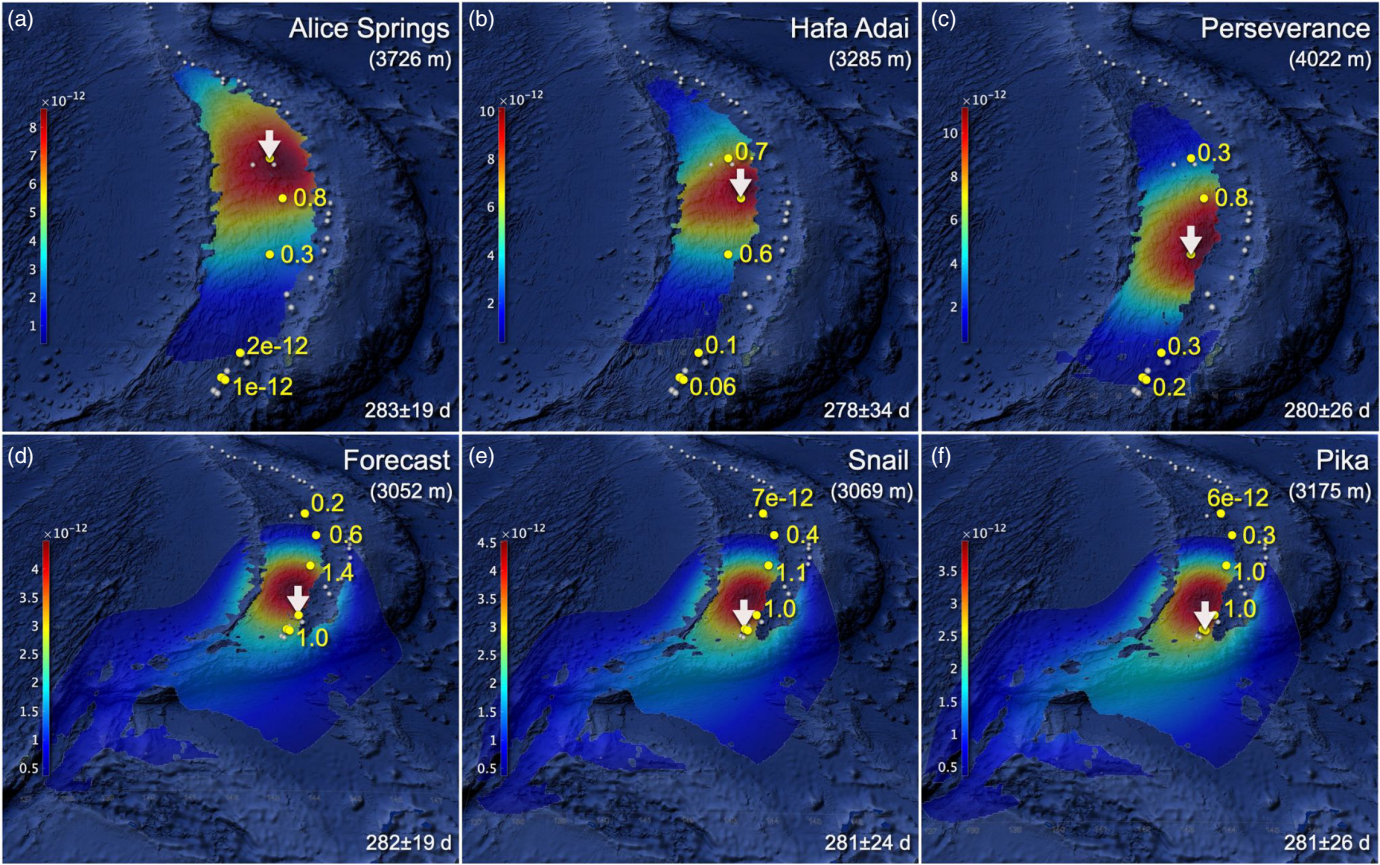
Modelled migration patterns for *Alviniconcha hessleri* in the Mariana Back‐Arc Basin. Colours indicate probability densities of water parcel displacement per unit area (square kilometres) from (a) Alice Springs, (b) Hafa Adai, (c) Perseverance, (d) Forecast, (e) Snail and (f) Pika (indicated with white arrows) at a dispersal depth of 3000 m. See caption of Figure 2 for more details. Map data: Google, SIO, NOAA, U.S. Navy, NGA, GEBCO, LDEO‐Columbia, NSF

For the Mariana back‐arc, modelled dispersal patterns in the northern and central basin (Alice Springs, Hafa Adai, Perseverance) were distinct from those in the southern end of the basin (Forecast, Snail, Pika) (Figure 3; Table S6). Particles from the northern and central Mariana back‐arc were mostly contained within the basin even for advection times of ~280 days and showed the highest dispersal probabilities around their initial positions. By contrast, particles from Forecast, Snail and Pika were not contained within the back‐arc basin, and some of them spread westward in the direction of the North Equatorial Current (while being blocked by bottom topography). Long‐term current observations at the southern end of the West Mariana Basin revealed mean southward flows (Yoshioka et al., 1988). However, Forecast, Snail and Pika are located on the east side of the basin, where compensatory northward flows promote transport towards the interior of the basin.

Particles from the northernmost sampling site Alice Springs did not reach Forecast, Snail or Pika (and vice versa). Even when accounting for stepping stone transport, connections among these sites are unlikely, that is, orders of magnitude smaller than Hafa Adai and Perseverance (Figure 3). Interestingly, Alice Springs and Forecast showed asymmetric directionality: northward connections from Forecast to Alice Springs are orders of magnitude larger than those into the other direction due to the northward dispersal trend described above. Overall probabilities for dispersal within the Mariana back‐arc increased with depth: larvae travelling at 3000 m were 20% more likely to arrive at another destination than those dispersing at the surface.

## 4 DISCUSSION

We explored the concept that a good understanding of larval transport pathways should reflect realized connectivity of vent metapopulations. Our data imply that closely related species exhibit contrasting migration patterns within and among back‐arc basins and that these patterns do not always correspond to expected dispersal patterns based on oceanographic conditions. Populations of *A*. *boucheti*, the species with the widest distribution in the Western Pacific, were genetically distinct among basins and gene flow occurred predominantly in an eastward direction. The observed genetic subdivision across basins is in agreement with previous genetic studies on other vent species (Kojima et al., 2000; Kyuno et al., 2009; Plouviez et al., 2019; Thaler et al., 2011, 2014). It also agrees with dispersal simulations by Mitarai et al. (2016) that indicate larval dispersal between Western Pacific back‐arc basins occurs rarely, that is, only once every 10,000s–100,000s of years. However, at least for *A. boucheti*, the directionality of our gene flow estimates differed from predictions of the biophysical model, suggesting that the Manus Basin might be a source instead of a sink site for dispersing larvae.

These scenarios are supported by a recent study by Plouviez et al. (2019) showing that gene flow between vent limpet populations occurs mainly from the Manus to the Lau Basin but not in the opposite direction. Thaler et al. (2014) suggested that the St. George's undercurrent might provide an occasional dispersal pathway for larvae out of the Manus Basin, but undiscovered vent sites, current anomalies and extended PLDs might be equally relevant (Plouviez et al., 2019). First, surveys for hydrothermal discharge based on oxidation‐reduction potential and turbidity indicate that active vent sites on fast‐to‐intermediate spreading centres are 3–6 times more frequent than previously thought (Baker et al., 2016), although nearly all verified sites are low discharge not favoured by *Alviniconcha*. Second, seasonal climatic events, such as the El Niño Southern Oscillation, typically enhance eastward currents (Wang et al., 2017), which could at least occasionally mediate larval transport from the Manus towards the Lau and North Fiji basins. Third, many marine invertebrate larvae can delay metamorphosis in the absence of suitable settlement cues (Pechenik, 1990), thereby increasing larval life and associated dispersal potential that might allow larvae to drift with occasional eastward currents for prolonged periods of time. Alternatively, the observed eastward‐directed gene flow in *A*. *boucheti* might reflect a historical colonization event, which could have occurred during past warming periods that led to abrupt reversals in oceanic currents (Nunes & Norris, 2006). *Alviniconcha kojimai* was the only other taxon that was sampled across back‐arc basins, but, in contrast to *A*. *boucheti*, populations of this species showed very limited gene flow (as expected based on dispersal predictions) despite lower genetic differentiation. This discrepancy might be partly explained by unsampled ghost populations, low sample sizes for some localities or limitations of the applied genetic markers, which highlights the importance of closing sampling gaps for accurate inference of population connectivity (Breusing et al., 2016). However, it is also possible that *A*. *kojimai* has different dispersal capabilities. Gary et al. (2020) detail the numerous possible strategies that a deep‐sea larva may deploy to affect dispersal. Their model assumes that all larvae ascend close to the surface but at differing speeds and durations that influence dispersal area. Nevertheless, not all vent larvae rise and closely related species have different life‐history strategies (Metaxas, 2011). For example, bottom‐hugging currents counter to prevailing transport can carry larvae of vent species along the strike of ridge features (Thomson et al., 2003). Furthermore, probabilities for dispersal in our own simulations were often highest at depth, suggesting that larvae of some *Alviniconcha* species likely disperse with deep‐water currents.

Population subdivision across back‐arc basins seemed unrelated to geographic distance. For example, the Niua South population of *A*. *boucheti* was less divergent from populations in the Manus Basin than from those in the nearby North Fiji Basin, while the reverse was true for the population at Kilo Moana. The differential genetic isolation of these sites may result from a combination of prevailing oceanographic and geomorphological conditions, historical demographic events and unknown ecological barriers. For example, the Lau and North Fiji basins have a two‐way connection through the North Fiji Passage that likely promotes gene flow to and from Kilo Moana. Simons et al. (2021) describe a strong north‐westerly outflow on the west side of the Lau Basin and an inflow south‐eastward to the central basin; both flow structures lie between about 1000 and 2000 m depth. Conversely, Niua South lies adjacent to the Tofua Passage, where westward deep‐water inflows to the Lau Basin (Simons et al., 2021) likely create significant barriers to dispersal into the site. Differences in dispersal depths or ontogenetic vertical migrations between populations could also play a role. Niua South is more similar in depth to sites in the Manus Basin than to either Kilo Moana or the North Fiji Basin vents. Larvae that are transported between Niua South and the Manus Basin would thus need to cross a smaller depth gradient than those dispersing towards Kilo Moana or the North Fiji Basin.

Additionally, habitat choice or differential adaptation of recruiting larvae to ecological conditions might exert strong control on connectivity patterns between populations, including physicochemical habitat properties, interspecies competition and facilitation, predation and the availability of compatible bacterial symbiont types (Beinart et al., 2012; Breusing, Johnson, et al., 2020; Breusing, Mitchell, et al., 2020; Lenihan et al., 2008; Micheli et al., 2002). Our analyses suggest that the presence of mitonuclear discordance in several *Alviniconcha* species could be the result of contrasting selective pressures acting on the mitochondrial *COI* (selective sweep or purifying selection) and the *ATPSb* genes (balancing selection), although other processes such as sex‐biased dispersal might contribute to these patterns (Toews & Brelsford, 2012). Both *COI* and *ATPSb* code for essential components of the mitochondrial respiratory chain, which likely imposes constraints on sequence evolution. At the same time, oxygen limitation at hydrothermal vents might select for certain variants of these loci that promote adaptation to hypoxic conditions, especially in species like *Alviniconcha* that occupy a high temperature, low oxygen niche (Podowski et al., 2010).

The effects of natural selection are probably also reflected in the observed genetic subdivision between populations of *A*. *boucheti* within the North Fiji Basin, where ocean circulation should promote widespread connectivity (Mitarai et al., 2016). By contrast, *A*. *strummeri* populations in the Lau Basin were well connected due to favourable current regimes, although gene flow seemed to be mainly southward. As probabilities were lower for dispersal at surface and 100 m than for dispersal at depth, larvae likely stay deep and may detect vent fluid plumes that rise to about 1700 m (Speer & Thurnherr, 2012). In the Mariana back‐arc, gene flow between *A*. *hessleri* populations largely corresponded to oceanographic conditions. Populations were partitioned between the northern and southern Mariana vent sites as a result of dispersal barriers that develop from the dominant flow regimes: weak southerly flows connect the northern sites to the central ones, but a northward inflow to the basin probably supports the observed gene flow from southern to central sites. It is likely that *A*. *hessleri* has near‐bottom larvae as dispersal probabilities diminished at shallower depths, a strategy also suggested for a squat lobster in the Manus Basin that shows significant differentiation between vent locations there (Thaler et al., 2014). Patterns of population structure in *A*. *hessleri* might have been further enhanced by historical fluctuations in hydrothermal activity that altered population dynamics within the region (Hidaka et al., 2015).

The asymmetry in gene flow patterns and low among‐basin connectivity even for taxa with long‐distance dispersal potential such as *Alviniconcha* (Warén & Bouchet, 1993) raise doubts about the resilience of Western Pacific vent ecosystems to disturbances from future deep‐sea mining activities. Although recent population dynamics models suggest mean recovery times of 5–140 years depending on the impacted region (Suzuki et al., 2018), these estimates are highly optimistic as community dissimilarities and interspecific differences in life‐history strategies and ecological requirements were not considered in the simulations. Especially, vent communities in back‐arc settings can be expected to recover slowly, as their relative stability compared to mid‐ocean ridge systems (Du Preez & Fisher, 2018) might have selected for life‐history strategies that are not adapted to sudden environmental changes. Seabed mining is predicted to significantly alter hydrothermal fluid flow regimes and substratum qualities (Van Dover, 2014), thereby changing basic ecological conditions that determine the habitability of the vent environment for associated animal species and their chemosynthetic symbionts. Consequently, even if a species has the potential to disperse to an impacted site, its habitat requirements might not be met to allow recruitment and maintain connectivity between populations. In addition, exclusion by long‐distance dispersing species that are able to adapt might prevent less resilient species from shifting their distributions under environmental change (Thompson & Fronhofer, 2019). For nearly all vent taxa, larval dispersal strategies and ecological niche characteristics are not well understood (Hilário et al., 2015), so that the recoverability of perturbed vent ecosystems remains difficult to predict.

Overall, the results from this study reveal several aspects that are relevant to regional, but also global conservation efforts: (a) Our data demonstrate that modelling of larval transport is a very useful tool to set hypotheses that should be tested for species of interest. Dispersal models can help guide field sampling for larvae and examination of the role of larval behaviour in affecting differential species responses to transport (Yearsley et al., 2020). However, to accurately address dispersal of deep‐sea vent larvae through modelling further comparative work on larval development and ecology is crucial. (b) Realized connectivity scenarios can vary – sometimes markedly – among taxa likely due to differences in life‐history strategies, demographic processes and ecological interactions, which highlights the need to identify the species of concern and target complementary genetic work. These assessments can in turn be used to improve and extend parameterizations of biophysical models for more realistic simulations of dispersal and connectivity patterns. (c) Metapopulation and metacommunity dynamics are critical features of island‐like habitats, such as hydrothermal vents (Mullineaux et al., 2018). In particular, mapping source–sink pathways can guide conservation management. For example, the Manus Basin – a past target of SMS extraction – would appear to be a sink based on generalized current models, yet it is an important source for several species studied. Similarly, Niua South has a dormant mine tenement despite remaining an important source site for *A*. *boucheti* and possibly other species. (d) The recent discovery of intermediate vent sites on the Mariana back‐arc has contributed to our understanding of gene flow dynamics, demonstrating the importance of exploration efforts for effective conservation.

Considering these aspects will be helpful to provide comprehensive guidelines for environmental management plans aimed at conserving biodiversity at hydrothermal ecosystems.

## CONFLICT OF INTEREST

The authors declare no conflict of interest.

## Supporting information

Fig S1

Tables S1–S6

## Data Availability

Sequence data for analysis were previously published in Johnson et al. (2015) and Breusing, Johnson, et al. (2020) under GenBank accession numbers KF467922–KF467955, KF467676–KF467896, MT148466–MT148633, MT148410–MT148463, MT148366–MT148407, MT148330–MT148357, MT148104–MT148273, MT148092–MT148099, MT149006–MT149209, MT148886–MT149003, MT148648–MT148825, MT148634–MT148645, MT147892–MT148091, MT147724–MT147889, MT147432–MT147663, MT147386–MT147429, MT131487–MT131555, MT131557–MT131574, MT131576, MT131578, MT131579, MT131582–MT131623, MT131625–MT131627, MT131629–MT131675, MT131680–MT131753 and MT131755–MT131779.
