# Supplementary figures and images for: Differential patterns of connectivity in Western Pacific hydrothermal vent metapopulations: A comparison of biophysical and genetic models
Source: Evol Appl. 2021 Dec 9;16(1):22–35. doi: 10.1111/eva.13326 (PMC9850011; doi:10.1111/eva.13326)

A

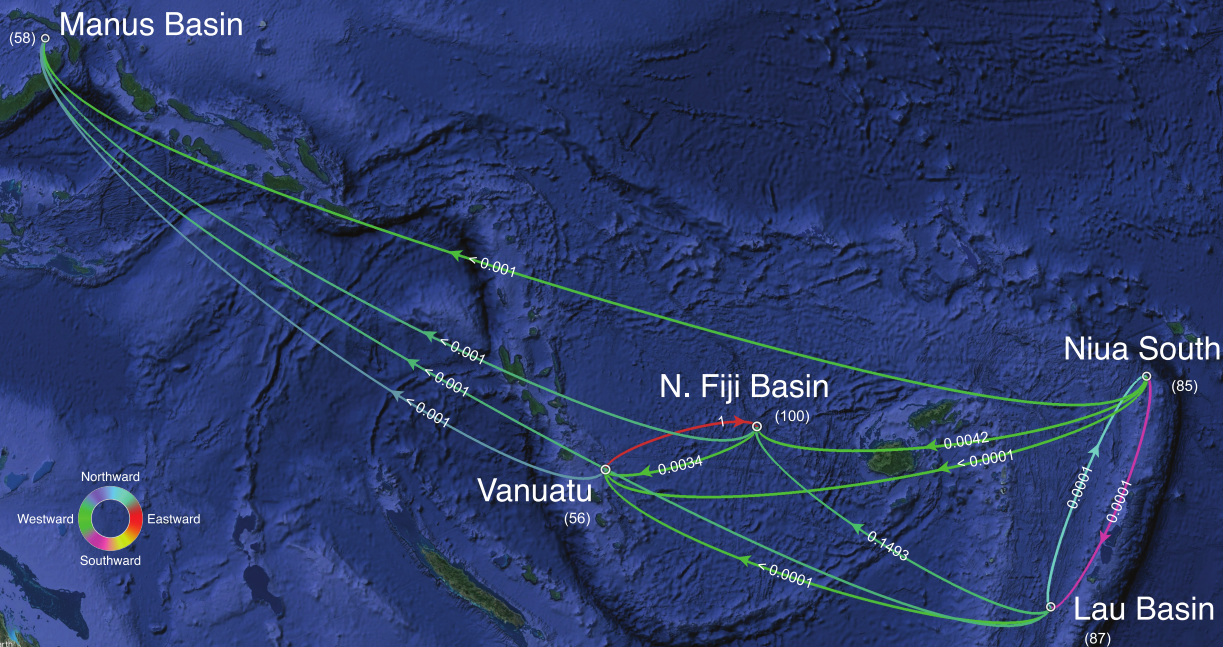

B

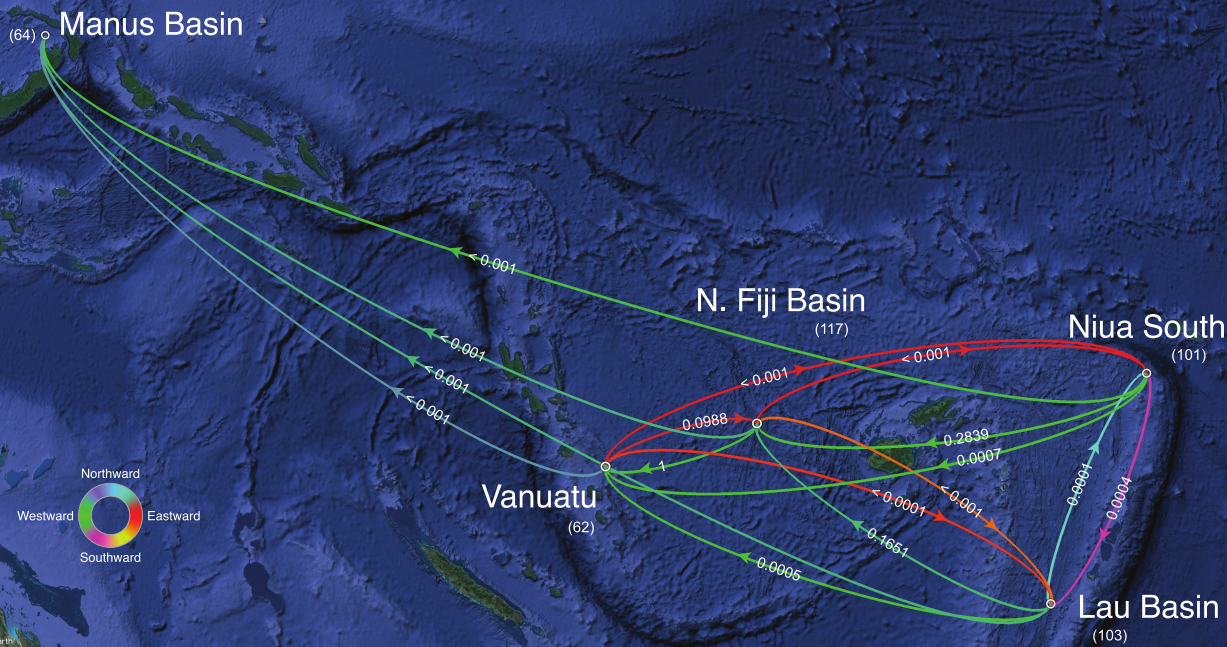

Supplement: Supplementary file 1 — Fig S1 [file EVA-16-22-s001.pdf]
