# Supplementary material for: Differential patterns of connectivity in Western Pacific hydrothermal vent metapopulations: A comparison of biophysical and genetic models
Source: Evol Appl. 2021 Dec 9;16(1):22–35. doi: 10.1111/eva.13326 (PMC9850011; doi:10.1111/eva.13326)
Supplement: Supplementary file 2 — Tables S1–S6 [file EVA-16-22-s002.docx]

**Supplemental Information for: Differential patterns of connectivity in Western Pacific hydrothermal vent metapopulations: A comparison of biophysical and genetic models**

Corinna Breusing, Shannon B. Johnson, Satoshi Mitarai, Roxanne A. Beinart, Verena Tunnicliffe

**Supplemental Tables**

**Table S1.** Analysis of Molecular Variance for mitochondrial and nuclear data at different levels of hierarchical subdivision. Subdivisions by regions and population for each species are shown in Tables 2 and 3.

|  | ***A. boucheti*** | | ***A. kojimai*** | | ***A. strummeri*** | | ***A. hessleri*** | |
| --- | --- | --- | --- | --- | --- | --- | --- | --- |
|  | % variation | *Φ*-statistics | % variation | *Φ*-statistics | % variation | *Φ*-statistics | % variation | *Φ*-statistics |
| **mtDNA** |  |  |  |  |  |  |  |  |
| Among regions | 0.7582 | 0.0076 | –0.7211 | –0.0072 | n.a. | n.a. | –1.5332 | –0.0153 |
| Among individuals within regions | –0.0139 | –0.0001 | 2.9760 | 0.0296 | –1.0295 | n.a. | 1.6788 | 0.0165 |
| Within individuals | 99.2557 | 0.0074 | 97.7451 | 0.0226 | 101.0295 | –0.0103 | 99.8545 | 0.0015 |
| **nDNA** |  |  |  |  |  |  |  |  |
| Among regions | 7.3937 | 0.0739 | –1.2010 | –0.0120 | n.a. | n.a. | 12.9514 | 0.1295 |
| Among sites within regions | 22.4997 | 0.2430 | 5.7845 | 0.0572 | 3.5588 | 0.0356 | 6.5910 | 0.0757 |
| Among individuals within sites | 40.9641 | 0.5843 | 43.6701 | 0.4577 | 72.2273 | 0.7489 | 48.0480 | 0.5972 |
| Within individuals | 29.1426 | 0.7086 | 51.7463 | 0.4825 | 24.2139 | 0.7579 | 32.4097 | 0.6759 |

**Table S2.** Diversity statistics and compound neutrality tests for each species and gene. π = nucleotide diversity (± standard deviation), *H* = haplotype diversity (± standard deviation), *S* = number of segregating sites, *D* = Tajima’s D, *H*_n_ = Fay and Wu’s H, *F* = Ewens-Watterson estimator, *P* = p-values for the different test statistics and the overall DHEW compound test (significant values are shown in bold).

| **Gene** | **Species** | **π** | ***H*** | ***S*** | ***D*** | ***P_D_*** | ***H*_n_** | ***P_H_*_n_** | ***F*** | ***P_F_*** | ***P*_DHEW_** |
| --- | --- | --- | --- | --- | --- | --- | --- | --- | --- | --- | --- |
| *COI* | *A. boucheti* | 0.0039±0.0025 | 0.8611±0.0216 | 50 | –2.0954 | **0.0019** | –5.3117 | **0.0005** | 0.1688 | **0.0026** | **0.0000** |
|  | *A. kojimai* | 0.0062±0.0036 | 0.8745±0.0242 | 64 | –2.2753 | **0.0005** | –7.1257 | **0.0000** | 0.1398 | **0.0001** | **0.0000** |
|  | *A. strummeri* | 0.0051±0.0031 | 0.8896±0.0366 | 35 | –2.1822 | **0.0017** | –4.3796 | **0.0016** | 0.1288 | **0.0052** | **0.0000** |
|  | *A. hessleri* | 0.0043±0.0027 | 0.7099±0.0547 | 44 | –2.3600 | **0.0002** | –5.7761 | **0.0001** | 0.3216 | **0.0000** | **0.0000** |
| *EF1a* | *A. boucheti* | 0.0223±0.0118 | 0.7008±0.0185 | 55 | 0.9061 | 0.8570 | –1.7601 | 0.0503 | 0.2700 | 0.1501 | 0.6628 |
|  | *A. kojimai* | 0.0051±0.0035 | 0.7145±0.0246 | 35 | –1.9343 | **0.0050** | –5.4415 | **0.0008** | 0.5218 | **0.0466** | **0.0031** |
|  | *A. strummeri* | 0.0089±0.0053 | 0.2349±0.0533 | 31 | –0.7432 | 0.2593 | –6.0796 | **0.0003** | 0.7674 | 0.1718 | 0.0585 |
|  | *A. hessleri* | 0.0105±0.0070 | 0.6563±0.0499 | 24 | –1.4307 | 0.0524 | 0.4961 | 0.6205 | 0.3360 | **0.0023** | 0.3315 |
| *ATPSa* | *A. boucheti* | 0.0383±0.0193 | 0.8739±0.0129 | 64 | 1.4441 | 0.9418 | –4.6735 | **0.0012** | 0.1894 | 0.5042 | 0.8373 |
|  | *A. kojimai* | 0.0124±0.0070 | 0.9539±0.0086 | 75 | –2.1750 | **0.0009** | –7.0895 | **0.0000** | 0.0846 | 0.1923 | **0.0272** |
|  | *A. strummeri* | 0.0288±0.0149 | 0.9860±0.0051 | 69 | –1.2732 | 0.0806 | –1.7611 | **0.0485** | 0.0411 | 0.0752 | **0.0051** |
|  | *A. hessleri* | 0.0011±0.0011 | 0.3279±0.0619 | 17 | –2.2924 | **0.0002** | 0.1943 | 0.4010 | 0.7082 | **0.0000** | 0.1262 |
| *ATPSb* | *A. boucheti* | 0.0504±0.0250 | 0.9287±0.0131 | 152 | –1.1705 | 0.1016 | –0.7408 | 0.1466 | 0.1254 | **0.0043** | **0.0138** |
|  | *A. kojimai* | 0.0182±0.0098 | 0.7341±0.0362 | 49 | –0.8869 | 0.1975 | –2.1429 | **0.0318** | 0.2444 | **0.0010** | **0.0313** |
|  | *A. strummeri* | 0.0128±0.0072 | 0.7597±0.0463 | 53 | –2.0099 | **0.0032** | –1.3583 | 0.0771 | 0.4783 | **0.0004** | **0.0064** |
|  | *A. hessleri* | 0.0166±0.0090 | 0.8175±0.0354 | 94 | –1.7707 | **0.0117** | –1.2754 | 0.0826 | 0.2212 | **0.0001** | **0.0057** |

**Table S3.** MLHKA test results for each species. lnL = log likelihood estimate of the model, *k* = selection parameter (*k* < 1 = reduction in polymorphism, *k* > 1 = excess in polymorphism), *P* = p-value of the log likelihood ratio test. Significantly higher likelihoods of selection models over neutral models are indicated in bold, while marginal significance is indicated in italics.

| **Species** | **Model** | **lnL** | ***k*** | ***P*** |
| --- | --- | --- | --- | --- |
| *A. boucheti* | Neutral | –34.0611 | 1.0000 |  |
|  | Selection on *COI* | –30.7940 | 0.2652 | **<0.050** |
|  | Selection on *EF1a* | –33.8786 | 0.7094 | >0.050 |
|  | Selection on *ATPSa* | –33.6304 | 0.6086 | >0.050 |
|  | Selection on *ATPSb* | –29.4440 | 3.6605 | **<0.010** |
| *A. kojimai* | Neutral | –28.0924 | 1.0000 |  |
|  | Selection on *COI* | –27.6206 | 0.6888 | >0.050 |
|  | Selection on *EF1a* | –28.0449 | 0.8827 | >0.050 |
|  | Selection on *ATPSa* | –26.8520 | 1.8904 | >0.050 |
|  | Selection on *ATPSb* | –28.0120 | 1.1887 | >0.050 |
| *A. strummeri* | Neutral | –29.6627 | 1.0000 |  |
|  | Selection on *COI* | –27.7258 | 0.4215 | **<0.050** |
|  | Selection on *EF1a* | –29.5341 | 0.7958 | >0.050 |
|  | Selection on *ATPSa* | –29.2710 | 1.4642 | >0.050 |
|  | Selection on *ATPSb* | –27.6070 | 2.4767 | **<0.050** |
| *A. hessleri* | Neutral | –30.8393 | 1.0000 |  |
|  | Selection on *COI* | –29.0882 | 0.2883 | *>0.050* |
|  | Selection on *EF1a* | –30.4016 | 0.4941 | >0.050 |
|  | Selection on *ATPSa* | –28.6842 | 0.2084 | **<0.050** |
|  | Selection on *ATPSb* | –23.9589 | 6.8873 | **<0.001** |

**Table S4.** Maximum likelihood estimates of population parameters from IMa3 analyses. Lower and upper 95% highest posterior densities are given in parentheses. Bolded values indicate significant migration under the log likelihood ratio test. • indicates poor estimates due to multiple peaks. *θ* = effective population size (4N_e_µ; µ = substitution rate per gene per generation); *t* = time of population splitting (Tµ; T = time since divergence in generations); *M* = migration rate (m/µ; m = proportion of immigrant gene copies in a population per generation).

|  | ***A. boucheti*** | ***A. kojimai*** | ***A. strummeri*** | ***A. hessleri*** |
| --- | --- | --- | --- | --- |
| *θ*_LB_ | 3.6 (0.3–12.7) | 28.7 (11.6–49.1) | – | – |
| *θ*_NS_ | 18.6 (0.3–133.5) | – | – | – |
| *θ*_TM_ | – | – | 33 (7.5–76.6) | – |
| *θ*_THM_ | – | – | 17.8 (3.8–39.4) | – |
| *θ*_NFB_ | 5.1 (1.9–8.5) | 11.4 (1.1–25.4) | – | – |
| *θ*_MB_ | 7.5 (2.9–12.1) • | 6.5 (1.6–10.1) | – | – |
| *θ*_VA_ | 1.4 (0.5–3.7) | – | – | – |
| *θ*_FS_ | – | – | – | 6.5 (3.1–10.3) |
| *θ*_HP_ | – | – | – | 105.1 (19.1–200) • |
| *θ*_IAB_ | – | – | – | 13.1 (4.7–21.5) |
| *θ*_A_ | 22.5 (11.5–35.5) | 11.4 (5.1–15.6) | 9.6 (4.6–13) | 6.4 (1.9–11.3) |
| *t*_LB+NS_ | – | – | – | – |
| *t*_TM+THM_ | – | – | – | – |
| *t*_LB+NS+NFB_ | 0.02 (0–0.3) | – | – | – |
| *t*_MB+VA_ | 0.3 (0.08–0.6) • | – | – | – |
| *t*_LB+NFB_ | – | 0.5 (0.2–1.1) | – | – |
| *t*_FS+HP+IAB_ | – | – | – | 1 (0.3–1.8) • |
| *t*_A_ | 0.3 (0.08–0.8) • | 0.6 (0.2–1) | 0.6 (0.1–1.3) | 1.2 (0.3–2.4) • |
| *M*_LB>NS_ | 2.1 (0–8.1) • | – | – | – |
| *M*_NS>LB_ | **8.4 (0.5–19.6)** | – | – | – |
| *M*_TM>THM_ | – | – | **6.1 (2–10)** | – |
| *M*_THM>TM_ | – | – | 4.3 (0–10) • | – |
| *M*_LB>NFB_ | **20.6 (1.2–50)** | 0.2 (0–1) | – | – |
| *M*_NFB>LB_ | **3.1 (0.5–6.3)** | 0.7 (0–2.4) | – | – |
| *M*_LB>MB_ | **12 (1–33.7)** | 0.6 (0–1.7) | – | – |
| *M*_MB>LB_ | **2.6 (0.6–4.9)** | 0.9 (0–2.8) | – | – |
| *M*_LB>VA_ | **11.6 (2–24)** | – | – | – |
| *M*_VA>LB_ | 4.8 (0–13.5) | – | – | – |
| *M*_NS>NFB_ | 5.6 (0–14.5) | – | – | – |
| *M*_NFB>NS_ | **3.5 (0.4–7.9)** | – | – | – |
| *M*_NS>MB_ | **15.6 (1.1–36.1)** | – | – | – |
| *M*_MB>NS_ | **0.7 (0–1.8)** | – | – | – |
| *M*_NS>VA_ | 14.5 (0–36) | – | – | – |
| *M*_VA>NS_ | **14.8 (1.9–32)** | – | – | – |
| *M*_NFB>MB_ | **0.7 (0.8–1.8)** | 1.3 (0–3.7) | – | – |
| *M*_MB>NFB_ | 0.7 (0–2) | 0.5 (0–1.8) | – | – |
| *M*_NFB>VA_ | 0.4 (0–1.2) | – | – | – |
| *M*_VA>NFB_ | **2.8 (0.3–7.7)** | – | – | – |
| *M*_MB>VA_ | **1.7 (0.8–2.8)** | – | – | – |
| *M*_VA>MB_ | **5.7 (0.2–12)** | – | – | – |
| *M*_FS>HP_ | – | – | – | 0.2 (0–0.1) |
| *M*_HP>FS_ | – | – | – | **28.8 (2.1–84.5)** |
| *M*_FS>IAB_ | – | – | – | 0.09 (0–0.9) |
| *M*_IAB>FS_ | – | – | – | 0.4 (0–1.3) |
| *M*_HP>IAB_ | – | – | – | **26.5 (0.5–85.2)** • |
| *M*_IAB>HP_ | – | – | – | 0.7 (0–1.9) |
| *M_ghost_* | *** | ** |  | * |

**Table S5.** Joint dispersal probabilities between vent localities in the Lau Basin and Tonga Volcanic Arc at different dispersal depths and corresponding mean pelagic larval durations (PLD) with standard deviation.

|  |  |  | **DESTINATION** | | | | | |
| --- | --- | --- | --- | --- | --- | --- | --- | --- |
| **SOURCE** | **0 m** | PLD | *Niua South* | *Kilo Moana* | *Tow Cam* | *Tahi Moana* | *ABE* | *Tu'i Malila* |
|  | *Niua South* | 19.71±0.99 | 1.0000 | 0.0000 | 0.0000 | 0.0000 | 0.0000 | 0.0000 |
|  | *Kilo Moana* | 22.40±2.10 | 0.0000 | 1.0000 | 0.9727 | 0.9062 | 0.8838 | 0.5566 |
|  | *Tow Cam* | 22.56±2.19 | 0.0000 | 1.0097 | 1.0000 | 0.9472 | 0.9257 | 0.6415 |
|  | *Tahi Moana* | 22.85±2.22 | 0.0000 | 0.9267 | 0.9717 | 1.0000 | 1.0044 | 1.0657 |
|  | *ABE* | 22.91±2.24 | 0.0000 | 0.8585 | 0.9210 | 0.9863 | 1.0000 | 1.1056 |
|  | *Tu'i Malila* | 23.79±2.49 | 0.0000 | 0.5314 | 0.6024 | 0.7011 | 0.7255 | 1.0000 |
|  | **100 m** | PLD | *Niua South* | *Kilo Moana* | *Tow Cam* | *Tahi Moana* | *ABE* | *Tu'i Malila* |
|  | *Niua South* | 21.79±1.10 | 1.0000 | 0.0000 | 0.0000 | 0.0000 | 0.0000 | 0.0000 |
|  | *Kilo Moana* | 26.06±1.25 | 0.0000 | 1.0000 | 0.9023 | 0.7205 | 0.6740 | 0.3756 |
|  | *Tow Cam* | 26.19±1.23 | 0.0000 | 1.0601 | 1.0000 | 0.9049 | 0.8790 | 0.5441 |
|  | *Tahi Moana* | 26.40±1.29 | 0.0000 | 0.9793 | 0.9906 | 1.0000 | 1.0016 | 0.7584 |
|  | *ABE* | 26.45±1.29 | 0.0000 | 0.9771 | 1.0037 | 1.0049 | 1.0000 | 0.7298 |
|  | *Tu'i Malila* | 27.19±1.49 | 0.0000 | 0.6295 | 0.7075 | 0.8094 | 0.8316 | 1.0000 |
|  | **500 m** | PLD | *Niua South* | *Kilo Moana* | *Tow Cam* | *Tahi Moana* | *ABE* | *Tu'i Malila* |
|  | *Niua South* | 94.28±4.34 | 1.0000 | 0.0000 | 0.0000 | 0.0000 | 0.0000 | 0.0000 |
|  | *Kilo Moana* | 82.74±3.85 | 0.0000 | 1.0000 | 0.9862 | 0.8601 | 0.8156 | 0.2156 |
|  | *Tow Cam* | 82.36±5.57 | 0.0000 | 1.0050 | 1.0000 | 0.9149 | 0.8843 | 0.4367 |
|  | *Tahi Moana* | 82.26±4.22 | 0.0000 | 0.9788 | 1.0264 | 1.0000 | 0.9803 | 0.4653 |
|  | *ABE* | 82.04±4.45 | 0.0000 | 0.9452 | 1.0165 | 1.0155 | 1.0000 | 0.4813 |
|  | *Tu'i Malila* | 81.14±4.61 | 0.0000 | 1.1645 | 1.3352 | 1.4999 | 1.5110 | 1.0000 |
|  | **1000 m** | PLD | *Niua South* | *Kilo Moana* | *Tow Cam* | *Tahi Moana* | *ABE* | *Tu'i Malila* |
|  | *Niua South* | 181.86±6.09 | 1.0000 | 0.0000 | 0.0000 | 0.0000 | 0.0000 | 0.0000 |
|  | *Kilo Moana* | 167.93±9.71 | 0.0000 | 1.0000 | 0.9656 | 0.8346 | 0.7921 | 0.2279 |
|  | *Tow Cam* | 167.85±11.69 | 0.0000 | 1.0073 | 1.0000 | 0.9174 | 0.8904 | 0.3604 |
|  | *Tahi Moana* | 168.42±12.53 | 0.0000 | 1.0131 | 1.0394 | 1.0000 | 0.9839 | 0.4642 |
|  | *ABE* | 168.35±12.58 | 0.0000 | 0.9783 | 1.0212 | 1.0096 | 1.0000 | 0.5026 |
|  | *Tu'i Malila* | 167.94±16.66 | 0.0000 | 0.8192 | 0.9563 | 1.1087 | 1.1412 | 1.0000 |
|  | **1500 m** | PLD | *Niua South* | *Kilo Moana* | *Tow Cam* | *Tahi Moana* | *ABE* | *Tu'i Malila* |
|  | *Niua South* | 227.40±19.53 | 1.0000 | 0.0000 | 0.0000 | 0.0000 | 0.0000 | 0.0000 |
|  | *Kilo Moana* | 225.13±11.35 | 0.0000 | 1.0000 | 0.8073 | 0.5718 | 0.5188 | 0.1477 |
|  | *Tow Cam* | 224.73±16.97 | 0.0000 | 1.1878 | 1.0000 | 0.7391 | 0.6800 | 0.2313 |
|  | *Tahi Moana* | 225.74±17.34 | 0.0000 | 1.3761 | 1.2290 | 1.0000 | 0.9466 | 0.3009 |
|  | *ABE* | 226.76±18.50 | 0.0000 | 1.4160 | 1.2867 | 1.0568 | 1.0000 | 0.3236 |
|  | *Tu'i Malila* | 226.51±21.38 | 0.0000 | 1.0088 | 1.1195 | 1.1843 | 1.1939 | 1.0000 |
|  | **2000 m** | PLD | *Niua South* | *Kilo Moana* | *Tow Cam* | *Tahi Moana* | *ABE* | *Tu'i Malila* |
|  | *Niua South* | n.a. | n.a. | n.a. | n.a. | n.a. | n.a. | n.a. |
|  | *Kilo Moana* | 252.24±15.66 | n.a. | 1.0000 | 0.9698 | 0.9082 | 0.8904 | 0.6085 |
|  | *Tow Cam* | 251.10±20.23 | n.a. | 1.0048 | 1.0000 | 0.9723 | 0.9628 | 0.7618 |
|  | *Tahi Moana* | 249.15±27.58 | n.a. | 0.9279 | 0.9551 | 1.0000 | 1.0150 | 1.2153 |
|  | *ABE* | 249.04±28.54 | n.a. | 0.8951 | 0.9291 | 0.9829 | 1.0000 | 1.2436 |
|  | *Tu'i Malila* | 240.24±49.04 | n.a. | 0.2255 | 0.2598 | 0.3282 | 0.3486 | 1.0000 |

**Table S6.** Joint dispersal probabilities between vent localities in the Mariana Back-Arc Basin at different dispersal depths corresponding mean pelagic larval durations (PLD) with standard deviation.

|  |  |  | **DESTINATION** | | | | | |
| --- | --- | --- | --- | --- | --- | --- | --- | --- |
| **SOURCE** | **0 m** | PLD | *Alice Springs* | *Hafa Adai* | *Perseverance* | *Forecast* | *Snail* | *Pika* |
|  | *Alice Springs* | 19.64±1.56 | 1.0000 | 0.6890 | 0.3519 | 0.0000 | 0.0000 | 0.0000 |
|  | *Hafa Adai* | 19.47±1.37 | 0.9865 | 1.0000 | 0.5275 | 0.0000 | 0.0000 | 0.0000 |
|  | *Perseverance* | 19.17±1.10 | 0.5133 | 0.5988 | 1.0000 | 0.3103 | 0.2150 | 0.2019 |
|  | *Forecast* | 18.94±0.84 | 0.0000 | 0.0000 | 0.4183 | 1.0000 | 0.9564 | 0.9308 |
|  | *Snail* | 18.93±0.79 | 0.0000 | 0.0000 | 0.3472 | 1.1504 | 1.0000 | 0.9614 |
|  | *Pika* | 18.93±0.78 | 0.0000 | 0.0000 | 0.3582 | 1.2011 | 1.0412 | 1.0000 |
|  | **100 m** | PLD | *Alice Springs* | *Hafa Adai* | *Perseverance* | *Forecast* | *Snail* | *Pika* |
|  | *Alice Springs* | 22.79±1.76 | 1.0000 | 0.7910 | 0.4823 | 0.0000 | 0.0000 | 0.0000 |
|  | *Hafa Adai* | 21.96±1.35 | 0.8252 | 1.0000 | 0.6205 | 0.0000 | 0.0000 | 0.0000 |
|  | *Perseverance* | 21.00±0.94 | 0.3146 | 0.4525 | 1.0000 | 0.4237 | 0.2726 | 0.2559 |
|  | *Forecast* | 20.20±0.78 | 0.0000 | 0.0000 | 0.1780 | 1.0000 | 1.0160 | 1.0030 |
|  | *Snail* | 20.11±0.77 | 0.0000 | 0.0000 | 0.0892 | 0.8474 | 1.0000 | 0.9674 |
|  | *Pika* | 20.10±0.78 | 0.0000 | 0.0000 | 0.0935 | 0.8484 | 1.0359 | 1.0000 |
|  | **500 m** | PLD | *Alice Springs* | *Hafa Adai* | *Perseverance* | *Forecast* | *Snail* | *Pika* |
|  | *Alice Springs* | 90.55±4.71 | 1.0000 | 0.6175 | 0.0000 | 0.0000 | 0.0000 | 0.0000 |
|  | *Hafa Adai* | 95.45±4.83 | 1.2885 | 1.0000 | 0.7357 | 0.0000 | 0.0000 | 0.0000 |
|  | *Perseverance* | 99.97±4.55 | 0.0000 | 0.6749 | 1.0000 | 0.8552 | 0.6615 | 0.6318 |
|  | *Forecast* | 105.39±5.99 | 0.0000 | 0.2144 | 0.5472 | 1.0000 | 0.9330 | 0.9160 |
|  | *Snail* | 107.47±5.86 | 0.0000 | 0.0000 | 0.3118 | 0.9270 | 1.0000 | 0.9877 |
|  | *Pika* | 107.69±5.81 | 0.0000 | 0.0000 | 0.3144 | 0.9347 | 1.0138 | 1.0000 |
|  | **1000 m** | PLD | *Alice Springs* | *Hafa Adai* | *Perseverance* | *Forecast* | *Snail* | *Pika* |
|  | *Alice Springs* | 183.42±3.09 | 1.0000 | 0.7304 | 0.3250 | 0.0000 | 0.0000 | 0.0000 |
|  | *Hafa Adai* | 183.48±3.79 | 0.6651 | 1.0000 | 0.8510 | 0.1543 | 0.0000 | 0.0000 |
|  | *Perseverance* | 184.13±5.66 | 0.1752 | 0.4756 | 1.0000 | 0.5691 | 0.3922 | 0.3782 |
|  | *Forecast* | 184.07±14.39 | 0.0000 | 0.2552 | 0.9326 | 1.0000 | 0.7559 | 0.7399 |
|  | *Snail* | 184.85±11.46 | 0.0000 | 0.0000 | 0.7013 | 1.1572 | 1.0000 | 0.9959 |
|  | *Pika* | 184.81±11.71 | 0.0000 | 0.0000 | 0.7059 | 1.1606 | 1.0023 | 1.0000 |
|  | **1500 m** | PLD | *Alice Springs* | *Hafa Adai* | *Perseverance* | *Forecast* | *Snail* | *Pika* |
|  | *Alice Springs* | 232.09±7.04 | 1.0000 | 1.0135 | 0.5979 | 0.0000 | 0.0000 | 0.0000 |
|  | *Hafa Adai* | 231.82±10.51 | 0.4125 | 1.0000 | 1.0849 | 0.1483 | 0.0000 | 0.0000 |
|  | *Perseverance* | 229.93±21.54 | 0.1383 | 0.5588 | 1.0000 | 0.3092 | 0.1701 | 0.1636 |
|  | *Forecast* | 229.86±16.58 | 0.0000 | 0.3138 | 0.8371 | 1.0000 | 0.8401 | 0.8283 |
|  | *Snail* | 229.17±13.63 | 0.0000 | 0.2305 | 0.7009 | 1.1154 | 1.0000 | 1.0003 |
|  | *Pika* | 228.79±16.03 | 0.0000 | 0.0000 | 0.6867 | 1.1288 | 0.9997 | 1.0000 |
|  | **2000 m** | PLD | *Alice Springs* | *Hafa Adai* | *Perseverance* | *Forecast* | *Snail* | *Pika* |
|  | *Alice Springs* | 261.41±7.24 | 1.0000 | 1.2765 | 0.7971 | 0.0990 | 0.0000 | 0.0000 |
|  | *Hafa Adai* | 260.96±12.46 | 0.4657 | 1.0000 | 0.6890 | 0.1070 | 0.0609 | 0.0584 |
|  | *Perseverance* | 259.87±16.09 | 0.2839 | 0.8158 | 1.0000 | 0.5725 | 0.4212 | 0.4131 |
|  | *Forecast* | 258.10±10.35 | 0.0000 | 0.2266 | 0.6498 | 1.0000 | 0.9466 | 0.9447 |
|  | *Snail* | 257.20±5.46 | 0.0000 | 0.0000 | 0.4093 | 0.9697 | 1.0000 | 1.0075 |
|  | *Pika* | 257.14±5.40 | 0.0000 | 0.0000 | 0.4190 | 0.9627 | 0.9927 | 1.0000 |
|  | **2500 m** | PLD | *Alice Springs* | *Hafa Adai* | *Perseverance* | *Forecast* | *Snail* | *Pika* |
|  | *Alice Springs* | 276.12±17.16 | 1.0000 | 1.0907 | 0.5646 | 0.0744 | 0.0000 | 0.0000 |
|  | *Hafa Adai* | 275.09±21.26 | 0.5630 | 1.0000 | 0.8126 | 0.2045 | 0.1139 | 0.1098 |
|  | *Perseverance* | 274.34±22.04 | 0.2762 | 0.6791 | 1.0000 | 0.6306 | 0.4177 | 0.4091 |
|  | *Forecast* | 273.63±16.67 | 0.0000 | 0.2560 | 0.7174 | 1.0000 | 0.9127 | 0.9089 |
|  | *Snail* | 273.64±10.24 | 0.0000 | 0.0000 | 0.4468 | 0.9718 | 1.0000 | 1.0014 |
|  | *Pika* | 273.48±11.75 | 0.0000 | 0.0000 | 0.4737 | 0.9792 | 0.9989 | 1.0000 |
|  | **3000 m** | PLD | *Alice Springs* | *Hafa Adai* | *Perseverance* | *Forecast* | *Snail* | *Pika* |
|  | *Alice Springs* | 282.68±18.86 | 1.0000 | 0.7798 | 0.3365 | 0.0000 | 0.0000 | 0.0000 |
|  | *Hafa Adai* | 277.75±34.39 | 0.7166 | 1.0000 | 0.6427 | 0.0989 | 0.0633 | 0.0596 |
|  | *Perseverance* | 279.82±26.40 | 0.3233 | 0.7649 | 1.0000 | 0.3044 | 0.1986 | 0.1892 |
|  | *Forecast* | 282.21±18.91 | 0.1887 | 0.6106 | 1.3858 | 1.0000 | 0.9849 | 0.9688 |
|  | *Snail* | 281.49±23.94 | 0.0000 | 0.3611 | 1.0774 | 0.9791 | 1.0000 | 0.9865 |
|  | *Pika* | 280.99±25.90 | 0.0000 | 0.3258 | 0.9807 | 0.9606 | 1.0116 | 1.0000 |

**Supplemental Figure Captions**

**Fig. S1** Modelled migration patterns between *Alviniconcha* populations across back-arc basins. Dispersal vectors are shown as joint transition probabilities for 100 generations normalized by the maximum value (from Vanuatu to North Fiji). Values were obtained from Mitarai et al. (2016). (A) Dispersal at 1000 m depth, (B) Dispersal at 500 m depth.
